# Integrative medicine during the intensive phase of chemotherapy in pediatric oncology in Germany: a randomized controlled trial with 5-year follow up

**DOI:** 10.1186/s12885-022-09703-0

**Published:** 2022-06-13

**Authors:** Georg Seifert, Sarah B. Blakeslee, Gabriele Calaminus, Farid I. Kandil, Andrea Barth, Toralf Bernig, Carl Friedrich Classen, Selim Corbacioglu, Jürgen Föll, Sven Gottschling, Bernd Gruhn, Claudia vom Hoff-Heise, Holger N. Lode, David Martin, Michaela Nathrath, Felix Neunhoeffer, Arnulf Pekrun, Beate Wulff, Tycho Zuzak, Günter Henze, Alfred Längler

**Affiliations:** 1 Department of Pediatrics, Division of Oncology and Hematology, Charité - Universitätsmedizin Berlin, Berlin, Germany; 2 Department of Pediatric Hematology and Oncology, University Children’s Hospital, Düsseldorf, Germany; 3 Department of Pediatric Hematology and Oncology, University Hospital Bonn, University of Bonn, Bonn, Germany; 4 Institute of Applied Analysis and Numerical Simulation, Research Group for Computational Methods for Uncertainty Quantification, University of Stuttgart, Stuttgart, Germany; 5 Department of Pediatrics, Martin Luther University Halle-Wittenberg, Halle, Germany; 6 Division of Pediatric Oncology, Hematology and Palliative Medicine Section, Department of Pediatrics and Adolescent Medicine, University Medicine Rostock, Rostock, Germany; 7 Department of Pediatric Hematology, Oncology and Stem Cell Transplantation, University Hospital Regensburg, Regensburg, Germany; 8 Center for Palliative Care and Pediatric Pain Medicine, Saarland University Medical Center, Homburg, Germany; 9 Department of Pediatrics, Jena University Hospital, Jena, Germany; 10 Department of Pediatric Hematology and Oncology, University Medicine, Greifswald, Germany; 11 Department of Hematology Oncology, University Children’s Hospital, Tübingen, Germany; 12 Department of Human Medicine, Faculty of Health, University Witten/Herdecke, Herdecke, Germany; 13 Pediatric Hematology and Oncology, Klinikum Kassel, Kassel, Germany; 14 Pediatric Oncology Center, Department of Pediatrics, Technische Universität München, Munich, Germany; 15 Department of Pediatric Cardiology, Pulmonology and Pediatric Intensive Care Medicine, University Children’s Hospital, Tübingen, Germany; 16 Department of Pediatric Hematology and Oncology, Hospital Bremen-Mitte, Bremen, Germany; 17 Department of Pediatric Hematology-Oncology, Pediatrics III, University Hospital of Essen, Essen, Germany; 18 Department of Integrative Pediatric and Adolescent Medicine, Gemeinschaftskrankenhaus Herdecke, Herdecke, Germany; 19 Centre for Integrative Medicine, University of Witten/Herdecke, Witten, Germany

**Keywords:** Pediatric oncology trial, Anthroposophic medicine, Mistletoe, Complementary cancer treatment, RCT, Randomized controlled trial

## Abstract

**Background:**

Integrative medicine is used frequently alongside chemotherapy treatment in pediatric oncology, but little is known about the influence on toxicity. This German, multi-center, open-label, randomized controlled trial assessed the effects of complementary treatments on toxicity related to intensive-phase chemotherapy treatment in children aged 1–18 with the primary outcome of the toxicity sum score. Secondary outcomes were chemotherapy-related toxicity, overall and event-free survival after 5 years in study patients.

**Methods:**

Intervention and control were given standard chemotherapy according to malignancy & tumor type. The intervention arm was provided with anthroposophic supportive treatment (AST); given as anthroposophic base medication (AMP), as a base medication for all patients and additional on-demand treatment tailored to the intervention malignancy groups. The control was given no AMP. The toxicity sum score (TSS) was assessed using NCI-CTC scales.

**Results:**

Data of 288 patients could be analyzed. Analysis did not reveal any statistically significant differences between the AST and the control group for the primary endpoint or the toxicity measures (secondary endpoints). Furthermore, groups did not differ significantly in the five-year overall and event-free survival follow up.

**Discussion:**

In this trial findings showed that AST was able to be safely administered in a clinical setting, although no beneficial effects of AST between group toxicity scores, overall or event-free survival were shown.

**Supplementary Information:**

The online version contains supplementary material available at 10.1186/s12885-022-09703-0.

## Introduction

### Background

Integrative medicine is used in up to 70% of adult oncology cases [1] and up to a third of pediatric oncology patients in Germany have been found to use some form of integrative therapy during cancer treatment [2]. Of pediatric patients in oncological treatment who also use integrative therapy, a broad survey with users found that anthroposophic supportive treatment (AST) was used by a third of patients in German pediatric oncological units [3]. Deriving from an alternative-holistic medical tradition developed by Steiner and Wegman in the 1920s that is well-established in Germany and in Europe, anthroposophic medicinal products (AMP) are used in cancer treatment for symptom management, to achieve a stable condition, to improve the tolerability of standard chemotherapy and improve the quality of life (QoL) [4]. AMP in pediatric oncology consist of a range of plant and animal-based tinctures, globules, extracts, injections, and compresses [3]. Mistletoe, given in oral and injectable form, is the most frequently used AMP in oncology and has demonstrated some benefit [5, 6]. Despite a century of AST for oncological patients such as mistletoe, the effect of treatments given in conjunction with standard chemotherapy is not well-studied in oncology and even less so in pediatric oncology. Evaluated exceptions have shown that in particular, children may benefit from AST interventions [3, 7]. The small body of research that does exist in pediatric oncology using AST has featured case studies highlighting safety [8–10] and quality of life for the young person [11, 12] rather than results of planned clinical trials. Parents of children suffering from intensive chemotherapy view AST as a possible means of assisting their child through difficult circumstances, but at the same time, the potential side effects of AST necessitate better monitoring clinical trials [2, 13]. This study is the first randomized controlled trial in pediatric oncology to systematically compare the toxicity of chemotherapy and survival in a 5-year follow up with or without an add-on AST intervention during intensive chemotherapy.

### Objective and hypothesis including 5-year follow up data

This study investigated the influence of the AST concept on the chemotherapy-associated toxicity in a randomized clinical trial undertaken at 12 German pediatric oncology clinics. The trial, consisting of an AST intervention, compared an application of AMP in pediatric oncology patients aged 1–18 undergoing standard chemotherapy to the control without AMP. A base of 8 AMP plus 11 on-demand, indication-related on-demand AMP were administered in the intervention group during the intensive chemotherapy phase. The trial tested the hypothesis that the AST would reduce the toxicity sum score for pediatric oncology patients undergoing standard treatment. The study documented long term changes with 5-year follow up data. The trial’s primary objective investigated the influence of the AST on the chemotherapy-associated toxicity measured by means of a toxicity sum score for hematology, mucositis, general condition and infection using NCI-CTC scales and the overall safety in terms of overall survival (OS) and event-free survival (EFS) until the end of the 5-year follow up. The secondary objective of the study was to investigate any decrease in chemotherapy-associated toxicity.

## Methods

### Trial design

This clinical trial investigated the effect of the AST for children undergoing chemotherapy combined with standard clinical care from 2005 to 2013 at 12 tertiary-level pediatric care units throughout Germany in a prospective, open-label, individually-randomized, controlled, national clinical study with parallel group design (Table 1: Inclusion & Exclusion Criteria Anthroposophic Supportive Therapy Study). The Institutional Review Board of Charité - Universitätsmedizin Berlin approved the trial as the responsible ethics committee (EA2/141/05). The study was registered at the European Union (EU) Drug Clinical Trials Register (EudraCT- No: 2004–002711-83) [14] before its commencement. All participants provided written informed consent in accordance to the Declaration of Helsinki [15].

**Table 1:** Inclusion & exclusion criteria anthroposophic supportive therapy study

**Inclusion Criteria:** • Age between 1 year and 18 years • Morphologically and/or immunologically • confirmed diagnosis of a following disease: ○ Hodgkin's disease (EuroNET-PHL-C1) ○ Acute lymphoblastic leukemia (ALL); ○ (ALL- BFM 2000; ALL-BFM 2000 incl. EsPhALL) ○ ALL (COALL 07-03) ○ Relapse of ALL (ALL-Rez BFM 2002) ○ Acute myeloid leukemia (AML) (AML-BFM 2004) ○ Nephroblastoma (SIOP 2001 / GPOH) ○ Germ cell tumors MAKEI 96 ○ Mature B-NHL / B-ALL (B-NHL - BFM 04; B- NHL BFM Rituximab) ○ Lymphoblastic lymphoma (until 06/2008 Euro-LB-02; from 07/2008 NHL-BFM 90) ○ Medulloblastoma / PNET or Ependymoma (HIT 2000) ○ Brain tumors-highly malignant (gliomas HIT-GBM-D; until 05/2009) ○ Neuroblastoma (NB 2004 and NB 2004 HR) ○ Osteosarcoma (EURAMOS 1) ○ Ewing's sarcoma (until 09/2009 EURO- E.W.I.N.G '99; from 10/2009 EWING 2008) ○ Rhabdomyosarcoma (CWS 2002P; until 6/2009) • Protocol-compliant therapy for the included diseases • Treatment in one of the study centers • Patients must be available during the treatment period and be able to comply with the study plan • Written consent for participation from the patient or the legal guardian	**Exclusion Criteria:** • Serious pre-existing/ co-existing psychiatric illness • Other existing serious medical condition that could interfere with the patient’s ability to receive trial- appropriate therapy • Any other condition or therapy that, in the opinion of the treating physician, could pose a risk to the patient or interfere with the objectives of the study • Absence of or incomplete informed consent form • Known allergies to any component of the study medications • Pregnancy or not using effective contraception (hormonal contraception, barrier) • Other experimental treatment during or within this study (including chemotherapeutic drugs or immunotherapies not listed in the protocol)

Three approved amendments were made to the original study protocol after commencement of the study and registered once the need for changes became apparent. These included necessary changes made to the inclusion of new and updated chemotherapy protocols, essential adjustments to administered treatments, necessary revision of study administration and modifications to inclusion/exclusion specifications.

### Study recruitment

Patients aged 1–18 with planned chemotherapy with a histologically and/or immunologically confirmed pediatric malignancy according to the current standardized treatment protocol were regarded eligible for trial recruitment and recruited from one of the 12 participating tertiary pediatric oncology units if the written consent was given. At the baseline, age, gender, educational background and familial situation was recorded for both groups. Sample size was determined based on the intention to decrease the NCI-CTC scale sum score of 2.1 obtained in the ALL-BFM-2000 study [16] by a clinically important difference of 10% to 1.9 in the intervention group (SD = 0.6, alpha = 0.05, Power = 80%).

### Study intervention

**Fig. 1:**
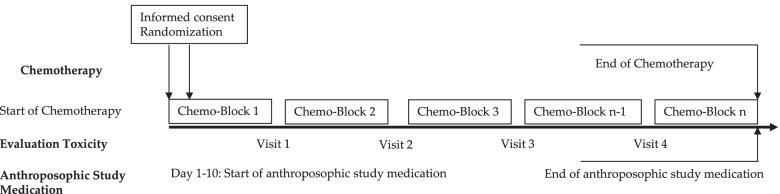
Trial design for the pediatric anthroposophic supportive treatment

Patients of the intervention group only were given the AST regimen as an add-on therapy to standard chemotherapy treatment (see Fig. 1: Trial Design for the Pediatric Anthroposophic Supportive Treatment).

The AST consisted of base AMP including Helixor®, and on-demand supplementary AMP given as needed for symptoms (summary in Table 2: Anthroposophic Supportive Treatment Base Medicinal Products and Supplementary Table). The control group received standard chemotherapy treatment without additional measures. Administration of the AST intervention and chemotherapy protocol were tailored for each type of pediatric malignancy included in the trial. This included both the base and the on-demand AMP, which were administered based on acute symptoms during intensive chemotherapy. The intervention group started the AST between the day of randomization and day 10 of the first chemotherapy cycle.

**Table 2:** Anthroposophic supportive treatment base medicinal products

Name, Dosage Form	Manufacturer	Ingredient	Indication	Application	Administration and dose
Helixor® A 0.1 mg, 1 mg, 5 mg, 10 mg, 20 mg, 50 mg,Solution for injection	Helixor Heilmittel GmbH	Aqueous fresh plant extract of *Viscum album* subspecies *abietis,* (fir mistletoe)	Malignant disease	Subcutaneous injection	2x week, dose increase dependent on skin reaction: 0.1 mg – 100 mg
Aurum/Prunus, Liquid dilution for injection	WALA Heilmittel GmbH	*Aurum metallicum dil*. D9; *Prunus spinosa e floribus et summitatibus ferm 33d* dil. D5 (HAB, Method 33d)	Aurum: Protection and sheath for the living organism Prunus: Strengthening of the immunological defense	Intravenous injection	1 ml ampule given before chemotherapy
Nux vomica D4, Solution for injection	Weleda AG	*Nux vomica Dil. D4*	For functional gastrointestinal disorders with nausea and/or vomiting	Intravenous injection	1 ml ampule given before chemotherapy
Cichorium planta tota 5%, Globules	WALA Heilmittel GmbH	*Cichorium intybus e planta tota ferm 33c* (chicory, HAB, Method 33c)	Stimulation of rhythmically mediated processes in the organism in terms of harmonization	Oral	< 4 years: 3 × 5 globules daily ≥ 4 years: 3 × 7 globules daily
Oxalis Folium Rh D4, Aqueous dilution	Weleda AG	*Oxalis, Folium Rh Dil. D4*	Stimulation and harmonization of metabolic processes, as well as excretory and digestive functions	Oral	< 4 years: 3 × 5 drops daily ≥ 4 years: 3 × 7 drops daily
Phosphorus D8, Globules	WALA Heilmittel GmbH	*Phosphorus dil. D8*	Strengthening of regenerative forces plus harmonization of sleep-wake-cycle	Oral	< 4 years: 5 globules daily in the morning ≥ 4 years: 10 globules daily in the morning
Phosphorus D30, Globules	WALA Heilmittel GmbH	*Phosphorus dil. D30*	Strengthening of regenerative forces plus harmonization of sleep-wake-cycle	Oral	< 4 years: 5 globules daily in the evening ≥ 4 years: 10 globules daily in the evening
Ratanhia comp., Solution	Weleda AG	*Myrrhae tinctura, Ratanhiae radix extractum fluidum, Aesculus, Cortex, ethanol. Decoctum Dil. D19, Argentum nitricum Dil. D14, Fluorit Dil. D9, Kieserit Dil. D19, Caryophylli floris aetheroleum, Eucalypti aetheroleum, Lavandulae aetheroleum, Menthae piperitae aetheroleum, Salviae officinalis aetheroleum*	Oral care during chemotherapy and in case of manifest mucosal lesions	Mouthwash	30 drops daily in 100 ml water

### Study randomization

Randomization was centrally allocated at the study headquarters (Charité) by trial administrators (GS, CHH) into intervention and control group by a computer supported standard operating procedure that used a combination of unique code identifier and abbreviation for the chemotherapy treatment.

## Results

### Sample description

**Fig. 2:**
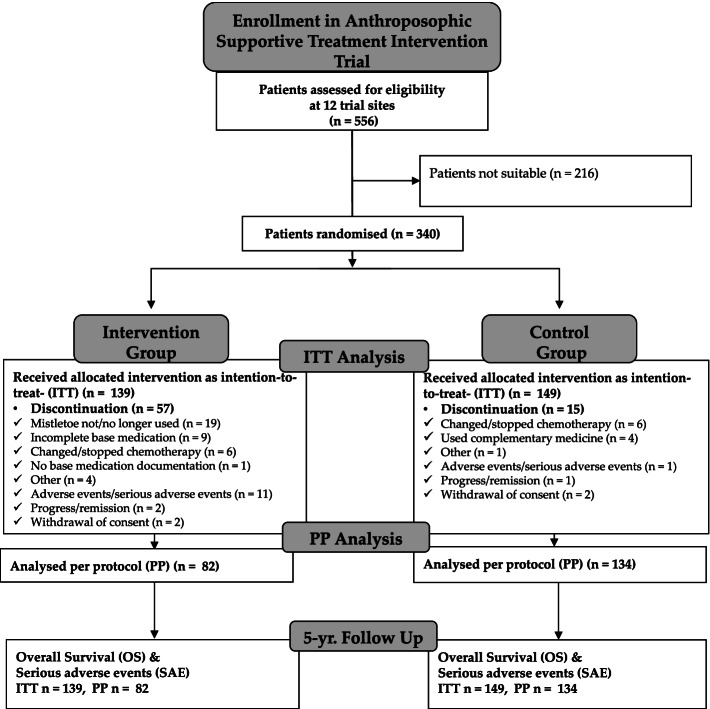
Anthroposophic supportive treatment trial consort chart

A total of 556 patients were screened for eligibility at the 12 trial sites between September 2005 and November 2013 (cf. Fig. 2: Anthroposophic Supportive Treatment Trial Consort Chart). Of these, 340 were found eligible, sorted into their diagnosis groups and then randomized. Reasons for ineligibility in the study were often due to necessary expediency of treatment or complexity of individual clinical cases that complicated study inclusion. After accounting for 52 (31 intervention, 21 control) dropouts, 288 patients were included in the intention-to-treat analysis (ITT), of which 216 patients completed all observational visits and administered at least 75% of the base study medications and could thus be included also in the per-protocol analysis (PP).

Demographic and clinical parameters of the patient groups can be found in Table 3: Baseline Characteristics. Baseline age, gender, educational background and familial situation in both groups were similar: About 62% of patients were male, average age in the intervention group was 8 years, while averaged 7.5 years in the control. The intervention group bodyweight averaged 35 kg and 32 kg in the control.

**Table 3:** Baseline characteristics

	ITT Population			PP Population		
	Intervention group	Control group	Total	Intervention group	Control group	Total
**Count (n)**	**139**	**149**	288	82	134	216
Sex: Female	51 (36.7%)	58 (38.9%)	109 (37.8%)	35 (42.7%)	50 (37.3%)	85 (39.4%)
Sex: Male	88 (63.3%)	91 (61.1%)	179 (62.2%)	47 (57.3%)	84 (62.7%)	131 (60.6%)
Weight [kg] Range	34.5 (8–110)	32.4 (8–92)	33.4 (8–110)	35.0 (10–92)	32.7 (9–110)	33.5 (9–110)
Study treatment duration						10.1 months (±8.10)

Patients suffering from an acute lymphoblastic leukemia (ALL) by far made up for the largest group with approximately 54% (154 / 288 ITT and 117 / 216 PP patients). Only a minor fraction had previous illnesses (< 15%).

### Primary outcome: the toxicity sum score (TSS)

**Fig. 3:**
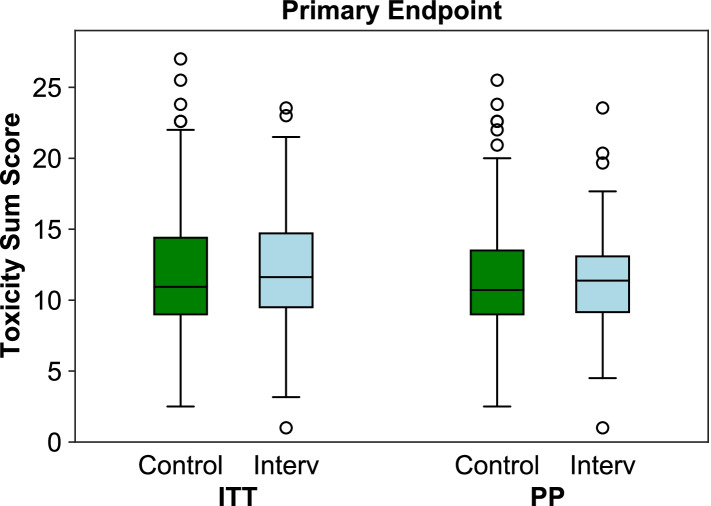
Toxicity Sum Score (TSS) primary outcome

The underlying data for the custom-defined Toxicity Sum Score (TSS) was available for 279 of the 288 ITT (96.9%) and for 208 of the 216 PP patients (96.3%). In the ITT analysis, the mean TSS was slightly higher in the intervention group (12.1 ± 3.92, median = 11.6) than in the control group (11.8 ± 4.54, median = 10.9), but slightly lower in the PP analysis (11.4 ± 3.58, median = 11.4 in the intervention group vs. 11.5 ± 4.33, median = 10.7 in the control group). Neither of these differences became statistically significant in the Mann-Whitney-U-Test, with *p* = 0.257 and *p* = 0.716 for the ITT and PP analysis, respectively (Fig. 3: Toxicity Sum Score (TSS) Primary Outcome).

### Secondary outcome: determination of chemotherapy-associated toxicity

Analysis of 43 additional toxicity criteria on the effect of AMP on other chemotherapy-related toxicity found a statistically significant reduction in number of diarrhea episodes in the intervention group (ITT *p* = 0.054; Mann-Whitney-U-test and PP- intervention group with *p* = 0.044, Mann-Whitney-U-test). However, there was no statistical significance for any other secondary outcome toxicity parameter in either the ITT or PP population.

### Adverse events

Adverse events were only recorded, and thus only analyzed, for the patients in the intervention group (initially 170 patients) who had self-administered at least one of the AMPs during the observational period (*n* = 163). All in all, 607 AE were documented in 123 patients, among them 532 (87.6%) with secured (*n* = 472), probable (*n* = 7), possible (*n* = 49) or undecidable (*n* = 4) causal relationship to the treatments.

The majority of the cases with secured causality, i.e. 447 cases observed in 105 patients, were accounted for by local inflammations at the injection site after subcutaneous administration of Helixor® A with a diameter of 5 cm and more. While local reactions from 2 to 5 cm in diameter are expected and even desired in mistletoe therapy, reactions with diameters of more than 5 cm are regarded as AE by the study protocol and thus documented. All other AE were related to the remaining products and were only observed in single cases.

### Serious adverse events

In contrast to AE, Serious Adverse Events SAE were recorded for the intervention and the control group. In total, 15 SAE in 11 patients were recorded for the main study period. Thorough evaluation by the study management and the Data and Safety Monitoring Committee (DSMC) showed that all SAE were related to chemotherapy-associated toxicities and not to the additional AST and were thus assessed as mislabeled records in the sense of the study protocol. Three of the misrepresented SAE had a lethal outcome with sepsis. Two were in the intervention group: one with fulminant sepsis with lethal outcome and the other with fulminant sepsis with absolute neutropenia and presumption diagnosis of intestinal perforation; these had no determined causal relationship with the intervention study medication. The third SAE occurred in the control group. According to the assessment of the study management, the cause for these SAE stemmed from the chemotherapy with which these patients were treated.

### Outcome of five-year follow up on event-free and overall survival & safety

Overall survival rates (OS) and events-free survival rates (EFS) were based on the intention-to-treat population (288 patients). For the analysis, data of patients in 12 groups with comparable chemotherapy were evaluated (upper half of Fig. 2). None of them showed a statistically significant difference according to the log-rank test, when Bonferroni correction was applied for multiple testing (alpha* = 0.0045). In the remaining six groups (ALL others, COALL Non-HR, Non-Hodgkin-Lymphoma, glioblastoma, germ cell tumor, nephroblastoma), no survival data analysis (log-rank test) could be applied because only one or no patient had died in the group.

Event-free survival rates (EFS) did not reveal any statistically significant differences between the two groups (lower half of Table 4).

**Table 4:** Secondary outcomes chemotherapy-associated toxicity parameter list

Secondary outcome parameter:	
Neutrophil granulocytes	
Neutropenia, number of days	
Red blood cell transfusion	
Transfusion of thrombocyte concentrates	
Fever, maximum temperature	
Antibiotics	
Antimycotics/antifungals	
Catheter infection	
Number of C-reactive protein values/measurements above the norm	
Number of C-reactive protein Values threefold above the norm	
Maximum CRP value	
Days with fever above 38,5 °C	
Nausea	
Emesis	
Stomatitis	
Number of days with stomatitis	
Abdominal pain/cramping	
Gastritis	
Obstipation	
Diarrhea episodes per day	
Pancreas ultrasonography/sonography	
Thrombosis	
Creatinine clearance	
Steroid diabetes	
Cushing syndrome	
Arrhythmia	
Cardiac function	
Echocardiography, left ventricular shortening fraction	
Pain	
Central neurotoxicity	
Fatigue	
Peripheral neurotoxicity	
Mood swings: depression	
Mood swings: anxiety	
Mood swings: euphoria	
General wellbeing	
Skin alterations	
Osteonecrosis	
Delay in onset of the last treatment block	
Hemoglobin	
Thrombocytes	
Leucocytes maximum value	
Leucocytes minimum value	
CRP maximum value	
Alpha lipase	
Glucose	
Aspartat amino transferase (AST, ASAT)	
Alanin amino transferase (ALT, ALAT)	
Bilirubin	
Creatinine	
Amylase	
Fibrinogen	
Antithrombin III (AT-III)	
Proteinuria	
prothrombin time (PTT)	

## Discussion

This randomized controlled clinical trial investigated the efficacy and safety of an anthroposophic supportive therapy concept consisting of 19 investigational medicinal products, applied as a base and on-demand therapy in addition to standard chemotherapy treatment in children with cancer. For the primary efficacy parameter, the toxicity sum score found no advantage for administration of the anthroposophic supportive therapy that could be demonstrated. Further NCI-CTC toxicity index scores to analyze secondary efficacy parameters only showed an advantage for the administration of the supportive therapy in the reduction of the toxicity index score for diarrhea in the PP-population. In the long term follow up, the explorative analysis of the data available for the 5-year follow up found no indications that efficacy of chemotherapy was influenced by AST. For long-term toxicities there were also no indications of an influence of AST. The AST-concept can be considered as safe in the long-term observation.

Trial findings confirmed that AST was able to be safely administered in a clinical setting. Overall, the analysis of AE including clinical experiences did not reveal any evidence of safety concerns with respect to the administration of the anthroposophic supportive therapy. Additionally, results of this study found no concerns of the compatibility of anthroposophic supportive therapy with the chemotherapy. This study demonstrated that the administration of the anthroposophic supportive therapy did not disrupt or delay therapy application in the intervention group, which is a crucial precondition for effective administration of chemotherapy.

This study showed the feasibility of conducting a high-quality, digitally-centralized randomized, scientific evaluation of an integrative therapy at multiple centers in the pediatric oncology setting. To our knowledge, there has only been one published comparable randomized study of an integrative therapy in pediatric oncology [17]. A strength of this first randomized controlled trial of a complementary anthroposophic treatment to be conducted within pediatric oncology demonstrated the safety of the intervention, the most critical overall outcome. This is comparable to findings in other randomized studies with adults [18, 19]. Additionally, the trial was conducted with a high methodological standard with centralized digital data management and inclusion of multiple centers, and provided long term follow up of the intervention effects.

### Limitations

Limitations, however, should also be mentioned. The complexity of the range of base and on-demand AMP necessitated clearer application guidelines at the study outset that may have permitted more extensive use in symptom treatment. This, on the other hand was difficult to tailor to individual participant tumor entities given the heterogeneity of malignancies included in the trial.

While other studies have reached the conclusion of a weak evidence base for single substance efficacy such as with viscum album (mistletoe) given in oncology trials with adults [5], marked quality of life factors have still been found to improve [18, 20]. As one of the success stories in oncology, adjustments to treatment dosages and schedules in pediatric oncology therapy has led simultaneously to an increase of survival and targeted therapeutics with better outcomes and resulting in less toxicity [21, 22]. One possible explanation for a lack in demonstrable gains in toxicity measures is the ceiling effect of targeted treatments and optimized symptom management.

However, studies that systematically research the effects of combined and comprehensive AST have been markedly absent. This study contributes significantly to this data gap whereby integrative treatments such as AST are in demand [7] especially in pediatric oncology. AST and other integrative treatments are often already being given by parents of children suffering from chronic conditions such as cancer in Germany as a possible means of assisting their child through difficult circumstances without specific effects being monitored within clinical trials [2, 13]. A singular known case report has previously raised the possibility of a connection between the promotion of tumor progression of a non-Hodgkin lymphoma at the subcutaneous injection site of viscum album therapy [23]. However, this study makes a strong case, along with other published findings, to demonstrate the safety of mistletoe injections in pediatric oncology [24, 25]. Where other areas of integrative medicine have shown efficacious results [26], facilitating a study design that investigates efficacy of combined AMP has provided an important lesson for the design of future research. Logistical and financial trade-offs must be carefully weighed and caution is needed in expediating hopeful outcomes for pediatric oncology patients, even if potential benefits to the quality of life may exist. While rigorous research is still needed for the application of comprehensive AST, a targeted approach, focusing for instance on one malignancy population or with a mixed-method design would potentially better capture effects of AMP.

## Conclusions

This study investigated an anthroposophic supportive therapy concept consisting of 19 medications as add-on therapy to standard treatment in children with cancer. For both the primary target criterion toxicity sum score and secondary target parameters, no advantage of using anthroposophic supportive therapy could be shown in this setting. Considering the previous clinical experience in the study population, it can be assumed that the anthroposophic supportive therapy can be applied safely. A key result is that there was no delay in therapy or reduction in therapy in the intervention group nor a statistical difference in 5-year survival as a result of the use of anthroposophic supportive therapy; an essential precondition for effective application of chemotherapy. Notable methodological and logistical lessons were demonstrated about the application of a supportive therapy concept in pediatric oncology that has important transferability for future research.

## Supplementary Information

**Additional file 1.**

## Data Availability

Datasets generated and/or analyzed during the current study or full study protocol are available upon request via the corresponding author.

## References

[1] Ernst E (2001). The current position of complementary/alternative medicine in cancer. Eur J Cancer.

[2] Längler A, Spix C, Seifert G, Gottschling S, Graf N, Kaatsch P (2008). Complementary and alternative treatment methods in children with cancer: a population-based retrospective survey on the prevalence of use in Germany. Eur J Cancer.

[3] Längler A, Spix C, Edelhäuser F, Martin DD, Kameda G, Kaatsch P (2010). Anthroposophic medicine in paediatric oncology in Germany: results of a population-based retrospective parental survey. Pediatr Blood Cancer.

[4] Kienle GS, Albonico HU, Baars E, Hamre HJ, Zimmermann P, Kiene H (2013). Anthroposophic medicine: an integrative medical system originating in europe. Glob Adv Health Med.

[5] Horneber MA, Bueschel G, Huber R, Linde K, Rostock M (2008). Mistletoe therapy in oncology. Cochrane Database Syst Rev.

[6] Steuer-Vogt MK, Bonkowsky V, Ambrosch P, Scholz M, Neiβ A, Strutz J (2001). The effect of an adjuvant mistletoe treatment programme in resected head and neck cancer patients: a randomised controlled clinical trial. Eur J Cancer.

[7] Stritter W, Rutert B, Eidenschink C, Eggert A, Längler A, Holmberg C (2020). Perception of integrative care in paediatric oncology-perspectives of parents and patients. Complement Ther Med..

[8] Zuzak TJ, Wasmuth A, Bernitzki S, Schwermer M, Längler A (2018). Safety of high-dose intravenous mistletoe therapy in pediatric cancer patients: a case series. Complement Ther Med.

[9] Seifert G, Rutkowski S, Jesse P, Madeleyn R, Reif M, Henze G (2011). Anthroposophic supportive treatment in children with medulloblastoma receiving first-line therapy. J Pediatr Hematol Oncol.

[10] Seifert G, Tautz C, Seeger K, Henze G, Laengler A (2007). Therapeutic use of mistletoe for CD30+ cutaneous lymphoproliferative disorder/lymphomatoid papulosis. J Eur Acad Dermatol Venereol.

[11] Kaestner J, Schlodder D, Preussler C, Gruhn B (2019). Supportive mistletoe therapy in a patient with metastasised neuroblastoma. BMJ Case Rep.

[12] Kameda G, Kempf W, Oschlies I, Michael K, Seifert G, Längler A (2011). Nodal anaplastic large-cell lymphoma ALK-1- with CD30+ cutaneous lymphoproliferation treated with mistletoe: spontaneous remission or treatment response?. Klin Padiatr.

[13] Gottschling S, Gronwald B, Schmitt S, Schmitt C, Längler A, Leidig E (2013). Use of complementary and alternative medicine in healthy children and children with chronic medical conditions in Germany. Complement Ther Med.

[14] EudraCT (European Union Drug Regulating Authorities Clinical Trials Database). Anthroposophic Supportive Therapy Trial Registration. EU Clinical Trials Register 2004. Report No.: Project-Code: 09–2004-PaedonkoChar.

[15] Association WM (2001). World medical Association declaration of Helsinki. Ethical principles for medical research involving human subjects. Bull World Health Organ.

[16] Schrappe M, Reiter A, Zimmermann M, Harbott J, Ludwig WD, Henze G (2000). Long-term results of four consecutive trials in childhood ALL performed by the ALL-BFM study group from 1981 to 1995. Berlin-Frankfurt-Münster Leukemia.

[17] Sencer SF, Zhou T, Freedman LS, Ives JA, Chen Z, Wall D (2012). Traumeel S in preventing and treating mucositis in young patients undergoing SCT: a report of the Children's oncology group. Bone Marrow Transplant.

[18] Tröger W, Galun D, Reif M, Schumann A, Stanković N, Milićević M (2013). Viscum album [L.] extract therapy in patients with locally advanced or metastatic pancreatic cancer: a randomised clinical trial on overall survival. Eur J Cancer.

[19] Augustin M, Bock PR, Hanisch J, Karasmann M, Schneider B (2005). Safety and efficacy of the long-term adjuvant treatment of primary intermediate- to high-risk malignant melanoma (UICC/AJCC stage II and III) with a standardized fermented European mistletoe (Viscum album L.) extract. Results from a multicenter, comparative, epidemiological cohort study in Germany and Switzerland. Arzneimittelforschung..

[20] Piao BK, Wang YX, Xie GR, Mansmann U, Matthes H, Beuth J (2004). Impact of complementary mistletoe extract treatment on quality of life in breast, ovarian and non-small cell lung cancer patients. A prospective randomized controlled clinical trial. Anticancer Res.

[21] Simone JV, Lyons J (1998). The evolution of cancer care for children and adults. J Clin Oncol.

[22] Adamson PC (2015). Improving the outcome for children with cancer: development of targeted new agents. CA Cancer J Clin.

[23] Hagenah W, Dörges I, Gafumbegete E, Wagner T (1998). Subcutaneous manifestations of a centrocytic non-Hodgkin lymphoma at the injection site of a mistletoe preparation. Dtsch Med Wochenschr.

[24] Stumpf C, Rosenberger A, Rieger S, Tröger W, Schietzel M (2000). Mistletoe extracts in the therapy of malignant, hematological and lymphatic diseases--a monocentric, retrospective analysis over 16 years. Forsch Komplementarmed Klass Naturheilkd.

[25] Kuehn JJ (1999). Favorable long-term outcome with mistletoe therapy in a patient with centroblastic-centrocytic non-Hodgkin lymphoma. Dtsch Med Wochenschr.

[26] Mühlenpfordt I, Stritter W, Bertram M, Ben-Arye E, Seifert G (2020). The power of touch: external applications from whole medical systems in the care of cancer patients (literature review). Support Care Cancer.

