# Supplementary material for: Integrative medicine during the intensive phase of chemotherapy in pediatric oncology in Germany: a randomized controlled trial with 5-year follow up
Source: BMC Cancer. 2022 Jun 13;22:652. doi: 10.1186/s12885-022-09703-0 (PMC9195372; doi:10.1186/s12885-022-09703-0)
Supplement: Supplementary file 1 — Additional file 1. [file 12885_2022_9703_MOESM1_ESM.docx]

**Supplementary Table 1:** **Anthroposophic supportive treatment concept - Composition, indication, application and dosage of anthroposophic medicinal products used**.

| **Name,**  **Dosage Form** | **Manufacturer** | **Ingredient** | **Intervention use** | **Study indication** | **Application** | **Administration and dose** |
| --- | --- | --- | --- | --- | --- | --- |
| Helixor^®^ A, 0.1 mg, 1 mg, 5 mg,  10 mg, 20 mg, 50 mg  Solution for injection | Helixor Heilmittel GmbH | Aqueous fresh plant extract of *Viscum album* subspecies *abietis,* (fir mistletoe) | Base medication | Malignant disease | Subcutaneous injection | 2x week,  dose increase dependent on skin reaction: 0.1 mg – 100 mg |
| Aurum/Prunus,  Liquid dilution for injection | WALA Heilmittel GmbH | *Aurum metallicum dil*. D9; *Prunus spinosa e floribus et summitatibus ferm 33d* dil. D5 (HAB, Method 33d) | Base medication | Aurum: Protection and sheath for the living organism  Prunus: Strengthening of the immunological defense | Intravenous injection | 1 ml ampule given before chemotherapy |
| Nux vomica D4, Solution for injection | Weleda AG | *Nux vomica Dil. D4* | Base medication | For functional gastrointestinal disorders with nausea and/or vomiting. | Intravenous injection | 1 ml ampule given before chemotherapy |
| Cichorium e planta tota 5%,  Globules | WALA Heilmittel GmbH | *Cichorium intybus e planta tota ferm 33c* (chicory, HAB, Method 33c) | Base medication | Stimulation of rhythmically mediated processes in the organism in terms of harmonization | Oral | < 4 years: 3x 5 globules daily  ≥ 4 years: 3x 7 globules daily |
| Oxalis Folium  Rh D4,  Aqueous dilution | Weleda AG | *Oxalis, Folium Rh Dil. D4* | Base medication | Stimulation and harmonization of metabolic processes, as well as excretory and digestive functions | Oral | < 4 years: 3x 5 drops daily  ≥ 4 years: 3x 7 drops daily |
| Phosphorus D8,  Globules | WALA Heilmittel GmbH | *Phosphorus dil. D8* | Base medication | Strengthening of regenerative forces plus harmonization of sleep-wake-cycle | Oral | < 4 years: 5 globules daily in the morning  ≥ 4 years: 10 globules daily in the morning |
| Phosphorus D30, Globules | WALA Heilmittel GmbH | *Phosphorus dil. D30* | Base medication | Strengthening of regenerative forces plus harmonization of sleep-wake-cycle | Oral | < 4 years: 5 globules daily in the evening  ≥ 4 years: 10 globules daily in the evening |
| Ratanhia comp., Solution | Weleda AG | *Myrrhae tinctura, Ratanhiae radix extractum fluidum, Aesculus, Cortex, ethanol. Decoctum Dil. D19, Argentum nitricum Dil. D14, Fluorit Dil. D9, Kieserit Dil. D19, Caryophylli floris aetheroleum, Eucalypti aetheroleum, Lavandulae aetheroleum, Menthae piperitae aetheroleum, Salviae officinalis aetheroleum* | Base medication | Oral care during chemotherapy and in case of manifest mucosal lesions | Mouthwash | 30 drops daily in 100 ml water |
| Argentum metallicum praeparatum D30, Solution for injection | Weleda AG | *Argentum metallicum praeparatum Dil. D30* | Interventional as needed | In case of fever from 38.5 ᵒC before intravenous application of antibiotics | Intravenous injection | < 4 years: 1 ampule daily  ≥ 4 years: 2 ampules daily |
| Bryophyllum 5%, Solution for injection | Weleda AG | *Aqueous extract of Kalanchoe pinnata, Folium rec.* | Interventional as needed | In case of restlessness, anxiety; awakening phase after sedation such as lumbar puncture, bone marrow puncture and anesthesia | Intravenous injection | < 4 years: 5 ml  ≥ 4 years: 10 ml |
| Aurum Valeriana Globuli velati,  Globules | WALA Heilmittel GmbH | *Aurum metallicum* dil. D5*, Camphora* dil. D3 *aquos., Crataegus laevigata/monogyna e foliis et fructibus* ferm 33d dil. D2*, Selenicereus grandiflorus ex herba* ferm 33d dil. D2*, Strophanthus kombe e semine* ferm 35b dil. D3*, Valeriana officinalis e radice* ferm 33c dil. D2 | Interventional as needed | In case of restlessness, anxiety; before interventions such as lumbar puncture, bone marrow puncture and anaesthesia | Oral | < 4 years: up to 2 hourly 5 globules  ≥ 4 years: up to 2 hourly 10 globules |
| Bryophyllum 50%, Powder to take orally | Weleda AG | *Pressed juice of Kalanchoe pinnata, Folium* | Interventional as needed | In case of adverse effects of steroids, sleep disorders | Oral | < 4 years: 3x ½ teaspoon daily  ≥ 4 years: 3x 1 teaspoon daily |
| Calendula Urtinktur, Mother tincture | Weleda AG | *Calendula officinalis* | Interventional as needed | In case of beginning mucositis | Oral | < 4 years: 3x 5 drops daily  ≥ 4 years: 3x 10 drops daily |
| Gentiana Magen Globuli velati (stomach remedy),  Globules | WALA Heilmittel GmbH | *Artemisia absinthium ex herba infusum, Gentiana lutea e radice decoctum, Strychnos nux-vomica e semine ferm 35b dil. D4, Taraxacum officinale e planta tota ferm 34c* | Interventional as needed | In case of absence of appetite | Oral | < 4 years: 3x 5 globules daily  ≥ 4 years: 3x 10 globules daily |
| Lachesis D8,  Liquid dilution | Weleda AG | *Lachesis Dil. D8* | Interventional as needed | In case of neutropenia | Oral | < 4 years: 3x 5 drops daily  ≥ 4 years: 3x 10 drops daily |
| Meteoreisen Globuli velati,  Globules | WALA Heilmittel GmbH | *Ferrum sidereum dil. D11 aquos., Phosphorus dil. D5, Quarz dil. D11 aquos.* | Interventional as needed | After infection or mucositis | Oral | < 4 years: 3x 5 globules  ≥ 4 years: 3x 10 globules |
| Nux vomica e semine D4,  Globules | WALA Heilmittel GmbH | *Strychnos nux-vomica e semine ferm dil. D4* | Interventional as needed | In case of nausea despite antiemesis | Oral | < 4 years: 3x 3 globules daily to hourly  ≥ 4 years: 3x 5 globules daily to hourly |
| Oxalis-Salbe 30%, Ointment | Weleda AG | *Ethanolic extract of Oxalis, Folium rec.* | Interventional as needed | In case of stomach aches, digestive disorders | Topical | On demand or regularly at noon as humid poultice |
| Solum Öl,  Oily embrocation | WALA Heilmittel GmbH | *Aesculus hippocastanum e semine* *LA* 25% *sicc*., *Equisetum arvense ex herba LA* 20%, *Lavandulae aetheroleum*, *Solum uliginosum*, *aqueous extract* | Interventional as needed | In case of restlessness and psychic lability | Topical | Inunction of single body parts (e.g. extremities/back) on demand, ideally in the evening |
